# The language of gait: interpreting emotional states through gait videos

**DOI:** 10.1007/s00415-025-13519-w

**Published:** 2025-11-18

**Authors:** Martina Putzolu, Elisabetta Sarasso, Elisa Canu, Andrea Gardoni, Elisa Ravizzotti, Susanna Mezzarobba, Silvia Basaia, Laura Avanzino, Massimo Filippi, Federica Agosta, Elisa Pelosin

**Affiliations:** 1 Department of Experimental Medicine, Section of Human Physiology, University of Genoa, Genoa, Italy; 2 Neuroimaging Research Unit, Division of Neuroscience, IRCCS San Raffaele Scientific Institute, Via Olgettina, 60, 20132 Milan, Italy; 3 Neurotech Hub, Vita-Salute San Raffaele University, Milan, Italy; 4 Department of Neuroscience, Rehabilitation, Ophthalmology, Genetics and Maternal Child Health, University of Genoa, Genoa, Italy; 5 IRCCS Ospedale Policlinico San Martino, Genoa, Italy; 6 Neurology Unit, IRCCS San Raffaele Scientific Institute, Milan, Italy; 7 Neurorehabilitation Unit, IRCCS San Raffaele Scientific Institute, Milan, Italy; 8 Neurophysiology Service, IRCCS San Raffaele Scientific Institute, Milan, Italy

**Keywords:** Gait, Emotion, Embodiment

## Abstract

**Introduction:**

Gait integrates sensorimotor and affective processes, serving both locomotor function and emotional expression. This embodiment framework has clinical relevance in neurodegenerative disorders and suggests potential for emotion-based modulation of gait.

**Objectives:**

This study developed and tested a range of emotion-specific gait videos to assess whether healthy individuals could correctly recognize different emotions, in order to identify standardized stimuli for future research on emotion recognition and embodied simulation in neurological patients.

**Methods:**

We created a video questionnaire featuring an actress walking with gait patterns meant to convey eight emotions: happiness, surprise, fear, anxiety, disgust, sadness, anger, and neutral. Her facial expressions were either visible or blurred to focus attention on body movements. Participants selected the recognized emotion from a list and rated its valence and intensity. Recognition accuracy, specificity, sensitivity, valence, and intensity scores were used to identify the most effective video for each emotion.

**Results:**

110 healthy subjects (aged 25–40 years) participated in the questionnaire. Most emotions—neutral, happiness, sadness, fear, and anger—were recognized with high accuracy (> 90%), specificity and sensitivity in blurred-face videos. Disgust and surprise were harder to identify, and anxiety was often confused with fear. Valence and intensity ratings aligned with the intended emotions for both blurred and visible faces, although they were generally higher for visible facial expressions.

**Conclusion:**

This study validates emotional gait videos as reliable stimuli for emotion recognition in healthy adults, laying the groundwork for their use in research on emotional embodiment in neurological disorders. These stimuli offer a tool for exploring emotion–gait interactions and developing emotion-based neurorehabilitation strategies.

**Supplementary Information:**

The online version contains supplementary material available at 10.1007/s00415-025-13519-w.

## Introduction

Human gait is a complex motor behavior that not only enables locomotion but also serves as a powerful channel for nonverbal emotional expression. Variations in gait, such as stride length, walking speed, arm swing, and postural alignment, can convey distinct emotional states, including happiness, sadness, anxiety, fear, and anger [1–3]. Increasing evidence suggests that observers can reliably infer emotional states based solely on the kinematic properties of walking [4–8]. This capacity for emotional recognition reflects a broader neurocognitive mechanism of embodiment, where emotions are both expressed and perceived through the body [9].

From a neurophysiological perspective, the ability to decode emotions through gait is supported by the mirror neuron system (MNS), primarily located in the fronto-parietal regions, as well as by limbic structures such as the amygdala and insula [10]. The MNS enables the internal simulation of observed motor acts, facilitating empathic understanding via embodied representations [11]. Functional imaging studies have shown that emotional gaits elicit distinct neural activation patterns, highlighting the integration of motor and affective processes [12].

This embodied perspective has meaningful clinical implications, particularly for neurodegenerative diseases like Parkinson’s disease (PD). Indeed, beyond classical motor symptoms such as bradykinesia and rigidity, PD patients often exhibit emotional and behavioral alterations, especially depression and anxiety, which contribute to gait disturbances [13–17]. Specifically, depression in PD is closely linked to the postural instability and gait disorder phenotype, and is associated with reduced gait speed, altered stride parameters, and increased variability, even in early stages and under dopaminergic treatment [13]. On the other hand, stressful and anxious situations can facilitate the occurrence of freezing of gait in PD, supporting the strong influence of the limbic system on motor behavior.

Recent research suggests that emotions influence gait not only in clinical populations but also in healthy individuals: negative emotions are typically associated with reduced speed, limited limb motion, and forward trunk flexion, whereas positive emotions correlate with faster walking, greater joint excursion, and upright posture [5, 18, 19]. This raises the question of whether emotional states could be intentionally employed to modulate and train spatio-temporal gait parameters, particularly in rehabilitation settings.

To date, physiotherapy interventions aimed at improving gait in neurological disorders, such as treadmill training, dual-task training, and multimodal mobility programs, have shown efficacy in enhancing spatio-temporal gait parameters [20]. Among these, Action Observation Training (AOT) has emerged as particularly promising due to its long-lasting effects. AOT leverages the activation of the MNS to facilitate motor learning through the observation of goal-directed actions [21–27]. However, the most effective ways to implement AOT remain under investigation, particularly regarding the content of the observed actions.

Given the complex interplay between emotion and gait, incorporating emotional content into AOT protocols may enhance outcomes. Observing gait patterns that embody specific emotions could not only reinforce motor learning but also evoke corresponding emotional states in the observer, a bottom-up embodiment effect. This approach may be especially valuable in PD, where emotional dysregulation and motor symptoms are intertwined.

To explore this possibility, recent studies propose using video stimuli that depict walking styles associated with distinct emotions (e.g., happiness, sadness, fear) [13]. These emotionally informed gait videos could be used to investigate how emotion modulates motor performance and perception in both healthy individuals and clinical populations. However, before these stimuli can be applied in neurological contexts, it is essential to assess their perceptual validity in healthy observers. Testing such stimuli ensures their emotional salience and discriminability across observers, thereby supporting both experimental rigor and clinical relevance.

Thus, the primary aim of the present study is to test whether healthy individuals can reliably recognize specific emotions from gait-based video stimuli. This process is a critical first step toward the selection of a set of standardized and emotionally salient gait videos. These videos will serve as experimental tools in future research exploring emotion recognition and embodied simulation in patients with neurological disorders such as PD. In this way, the study aims at establishing a robust methodological foundation for future applications in affective-motor rehabilitation.

## Materials and methods

### Inclusion criteria

We included healthy young adults ranging from 25 to 40 years of age in order to include subjects in their full emotional maturity (≥ 25 years old) [28] and without any potential influence of aging on emotional processes. Subjects were recruited by word of mouth among institute personnel and their acquaintances and they were excluded after a careful anamnesis if their medical history indicated any major neurological or psychiatric disorder, or substance abuse, which could interfere with cognitive processing.

### Questionnaire development

**Fig. 1:**
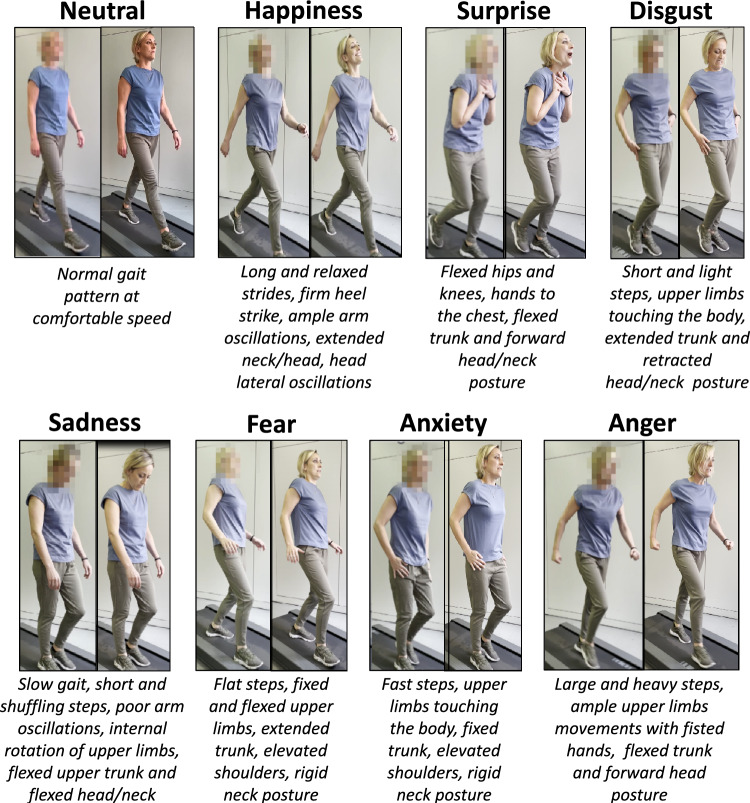
Representation and description of the gait patterns associated with each emotion

A questionnaire aimed at assessing the possibility to recognize emotions transmitted through body movements during gait was developed. The questionnaire was administered online and contained videos of an actress walking with different gait patterns according to specific emotions (happiness, surprise, fear, anxiety, disgust, sadness, anger, and neutral). The actress was instructed by an expert of gait analysis to walk on a treadmill while embodying these emotions, according to the gait patterns previously described in the literature [1, 5, 6, 18], and her facial expressions in the videos were intentionally blurred to promote emotion recognition through body movements (bottom-up embodiment). In total 16 videos were recorded, two videos for each emotion with two levels of body movement exaggeration according to the hypothesis that more exaggerated body movements would produce more accurate emotion classification and higher rating of emotional intensity [4]. After the observation of blurred-face videos, participants were shown the videos including facial expressions for the first time, to assess differences in emotion recognition and interpretation. Videos lasted approximately 6 s each and were presented once, in a randomized order for both blurred and facial expression videos. Participants first viewed all the videos with blurred faces and were not allowed to see the versions showing facial expressions beforehand. A graphical representation of the proposed emotional gait videos with the description of the relative gait features is presented in Fig. 1. All the videos are available at this link https://forms.office.com/e/B6w0755Jr8.

After the observation of each video participants were asked to select the emotion they believe the actress is experiencing from a list of emotions (happiness, surprise, fear, anxiety, disgust, sadness, anger, and neutral). Then participants were asked to rate the valence, defined as the pleasantness or unpleasantness of the emotion, perceived by the actress on scale ranging from 1 to 5 (1 = extremely positive, 2 = positive, 3 = neutral, 4 = negative and 5 = extremely negative) and the intensity of the emotion perceived by the actress on a scale ranging from 0 to 10 (0 = no intensity, 10 = maximal intensity).

### Statistical analysis

Statistical analyses were performed using IBM SPSS Statistics version 23. The significance level was set at *p* < 0.05. Descriptive statistics (percentage, frequency, mean and standard deviation) were used to describe the sample characteristics and the performance at the questionnaire. The normality of the results was assessed using the Shapiro–Wilk test. Considering the non-normal distribution of our data, comparisons between blurred-face videos and videos showing facial expressions were assessed using Mann–Whitney test for continuous variables, while categorical variables were analyzed using Chi-square tests.

To identify the most effective video for each emotion (the video achieving the highest percentage of emotion agreement), the accuracy, the mean value of valence and intensity, the sensitivity, specificity and F1 score were calculated. Inter-rater agreement was assessed using Fleiss’ Kappa (κ). For each stimulus, individual agreement indices (P_i_) were computed to quantify the level of consistency among participants’ responses. These steps allow for the selection of videos based on data from a healthy population. The selected videos will subsequently be presented to a more extended population of healthy subjects (in order to stratify results according to age) and to subjects with PD in future studies.

## Results

We recruited 110 healthy subjects by word of mouth among institute personnel and their acquaintances. The sample included 46 men and 64 women with a mean age of 30.64 ± 4.05 (range 25–40) and mean years of education 18.39 ± 2.74 (range 12–26). All the subjects underwent the complete questionnaire and results are summarized in Table 1.

**Table 1:** Emotional gait pattern recognition and interpretation in a population of 110 healthy adults during the observation of different walking conditions with blurred face

	Blurred-face gait videos			Facial expression gait videos			Blurred versus facial expression		
	Correct answers (%)	Valence (1/2/3/4/5)	Intensity (mean ± std)	Correct answers (%)	Valence (1/2/3/4/5)	Intensity (mean ± std)	*p* correct answers	*p* valence	*p* intensity
*Neutral*									
Neutral 1	98	3/4/99/3/1	3.17 ± 2.67	91	2/1/97/9/1	3.71 ± 2.95	**0.03**	0.29	0.17
Neutral 2	88	3/5/95/6/3	3.52 ± 2.70	94	2/1/99/8/0	3.48 ± 2.86	0.24	0.18	0.81
*Happiness*									
Happiness 1	66	35/50/23/1/1	5.95 ± 2.32	100	78/27/0/4/1	8.23 ± 1.17	** < 0.001**	** < 0.001**	** < 0.001**
Happiness 2	91	65/29/8/5/3	7.45 ± 1.60	100	87/15/0/4/4	8.54 ± 1.15	**0.002**	**0.003**	** < 0.001**
*Surprise*									
Surprise 1	85	15/60/18/16/1	6.31 ± 1.60	95	55/50/0/3/2	7.98 ± 1.17	**0.02**	** < 0.001**	** < 0.001**
Surprise 2	85	48/31/3/20/8	8.10 ± 1.40	93	86/14/0/4/6	8.92 ± 0.94	0.09	** < 0.001**	** < 0.001**
*Fear*									
Fear 1	84	0/4/15/63/28	7.50 ± 1.51	86	0/0/9/82/19	7.40 ± 1.29	0.71	**0.02**	0.51
Fear 2	91	0/0/21/68/21	7.44 ± 1.25	95	0/0/3/68/39	8.08 ± 1.00	0.29	** < 0.001**	** < 0.001**
*Anxiety*									
Anxiety 1	85	1/0/4/65/40	7.62 ± 1.50	89	0/1/10/72/27	7.55 ± 1.27	0.55	0.11	0.42
Anxiety 2	60	0/1/6/71/32	7.61 ± 1.43	75	0/1/1/82/26	7.60 ± 1.19	**0.03**	0.17	0.57
*Disgust*									
Disgust 1	38	0/2/24/77/7	6.55 ± 1.35	91	0/0/5/76/29	7.88 ± 1.17	** < 0.001**	** < 0.001**	** < 0.001**
Disgust 2	45	0/0/11/70/29	7.35 ± 1.54	95	0/1/6/65/38	8.04 ± 1.33	** < 0.001**	0.28	**0.001**
*Sadness*									
Sadness 1	87	0/0/23/74/13	6.20 ± 1.83	97	0/0/16/82/12	6.59 ± 1.53	**0.01**	0.43	0.16
Sadness 2	100	1/0/1/44/64	8.11 ± 1.40	100	0/0/2/50/58	7.87 ± 1.49	1.00	0.57	0.19
*Anger*									
Anger 1	92	0/1/14/62/33	7.11 ± 1.57	99	0/0/4/60/46	7.53 ± 1.35	**0.02**	**0.03**	0.07
Anger 2	98	0/0/1/18/91	8.62 ± 0.87	98	0/0/0/15/95	9.22 ± 0.75	1.00	0.51	** < 0.001**

*p* values refer to chi-square test for Correct answers and Valence, and to Mann–Whitney test for Intensity

Valence score ranged from 1 to 5 (1 = extremely positive, 2 = positive, 3 = neutral, 4 = negative and 5 = extremely negative). Intensity ranged from 0 to 10 (0 = no intensity, 10 = maximal intensity)

### Emotion recognition

**Fig. 2:**
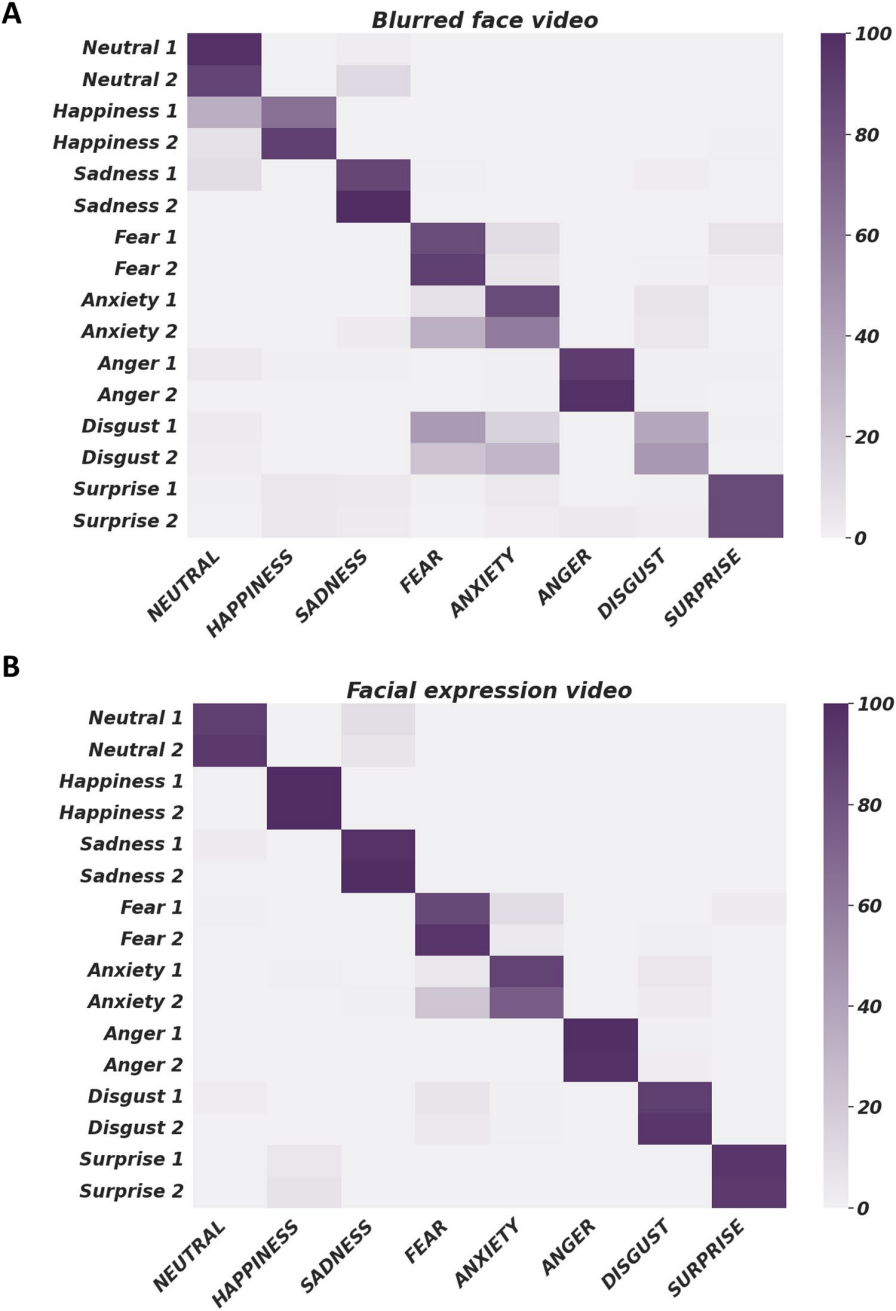
Performance of emotion recognition both in blurred-face videos (**A**) and in videos showing facial expressions (**B**). Color bar represents the percentage of the answers from 0 to 100

Performance of emotion recognition both in blurred-face videos and in videos showing facial expressions is reported in Fig. 2. Values of specificity, sensitivity, F1 score and P_i_ of blurred-face videos are reported in Supplementary Table S1.

Overall, the Fleiss’ Kappa analysis showed a κ of 0.68 (CI 95%: 0.65–0.72) representing a substantial level of agreement among participants in recognizing the emotional content of the videos.

Among the observed emotional gait videos with blurred face, the most frequently recognized conditions were neutral, happiness, sadness, fear and anger in which at least one video showed a mean accuracy exceeding 90% (ranging from 91 to 100%), specificity and sensitivity exceeding 0.9 (ranging from 0.91 to 1), F1 score ranging from 0.6 to 0.97 and P_i_ ranging from 0.83 to 1 (Supplementary Table S1). In contrast, surprise and disgust were more challenging to identify through observation of the gait pattern, with a mean accuracy < 90%. Surprise videos showed similar metrics of specificity (0.99), sensitivity (0.85), F1 score 0.87 and P_i_ (ranging from 0.72 to 0.73). Disgust videos showed the worse performance, with low sensitivity, F1 score, and P_i_ (Supplementary Table S1).

Anxiety was frequently confused with fear and vice-versa, supporting the common nature of these emotions. In this context, if we consider that both answers could be considered correct for each video, we identified effective videos also for fear/anxiety (exceeding 90% accuracy). Also in the case of anxiety, there was a video showing a good performance (specificity 0.95, sensitivity 0.85, F1 score 0.67 and P_i_ 0.74).

The detailed results are presented below:

#### Neutral

Video *Neutral 1* blurred was correctly recognized by 98% of participants (specificity 0.96, sensitivity 0.98, F1 score 0.75, P_i_ 0.96). In this video, neutral was confused with sadness (2%). The same video showing facial expressions was correctly recognized by 91% of participants, while 9% confused neutral with sadness. Blurred video was correctly recognized significantly more frequently than the one showing facial expressions (*p* = 0.03).

Video *Neutral 2* blurred was correctly recognized by 88% of participants (specificity 0.96, sensitivity 0.88, F1 score 0.70, P_i_ 0.79). In this video, neutral was confused with sadness (12%). The same video showing facial expressions was correctly recognized by 94% of participants, while 6% confused neutral with sadness.

#### Happiness

Video *Happiness 1* blurred was correctly recognized by 66% of participants (specificity 0.99, sensitivity 0.66, F1 score 0.75, P_i_ 0.55). In this video, happiness was confused with neutral (34%). The same video showing facial expressions was correctly recognized by 100% of participants. Blurred video was correctly recognized significantly less frequently than the one showing facial expressions (*p* < 0.001).

Video *Happiness 2* blurred was correctly recognized by 91% of participants (specificity 0.99, sensitivity 0.91, F1 score 0.90, P_i_ 0.83). In this video, happiness was confused with neutral (8%) or surprise (1%). The same video showing facial expressions was correctly recognized by 100% of participants. Blurred video was correctly recognized significantly less frequently than the one showing facial expressions (*p* = 0.002).

#### Surprise

Video *Surprise 1* blurred was correctly recognized by 85% of participants (specificity 0.99, sensitivity 0.85, F1 score 0.87, P_i_ 0.73). In this video, surprise was confused with happiness (5%), anxiety (4%), sadness (4%), disgust (1%), Fear (1%) or neutral (1%). The same video showing facial expressions was correctly recognized by 95% of participants, while 5% confused surprise for happiness. Blurred video was correctly recognized significantly less frequently than the one showing facial expressions (*p* = 0.02).

Video *Surprise 2* blurred was correctly recognized by 85% of participants (specificity 0.99, sensitivity 0.85, F1 score 0.87, P_i_ 0.72). In this video, surprise was confused with happiness (5%), anger (4%), sadness (3%), anxiety (2%) or disgust (2%). The same video showing facial expressions was correctly recognized by 93% of participants, while 7% confused surprise for happiness.

#### Fear

Video *Fear 1* blurred was correctly recognized by 84% of participants (specificity 0.93, sensitivity 0.84, F1 score 0.57, P_i_ 0.71). In this video, fear was confused with anxiety (10%) or surprise (6%). The same video showing facial expressions was correctly recognized by 86% of participants, while fear was confused for anxiety (10%), surprise (3%) or neutral (1%).

Video *Fear 2* blurred was correctly recognized by 91% of participants (specificity 0.93, sensitivity 0.91, F1 score 0.60, P_i_ 0.83). In this video, fear was confused with anxiety (6%), surprise (2%) or disgust (1%). The same video showing facial expressions was correctly recognized by 95% of participants, while fear was confused for anxiety (4%), or disgust (1%).

#### Anxiety

Video *Anxiety 1* blurred was correctly recognized by 85% of participants (specificity 0.95, sensitivity 0.85, F1 score 0.67, P_i_ 0.74). In this video, anxiety was confused with fear (8%) or disgust (6%). The same video showing facial expressions was correctly recognized by 89% of participants, while anxiety was confused for fear (5%), disgust (5%) or happiness (1%).

Video *Anxiety 2* blurred was correctly recognized by 60% of participants (specificity 0.95, sensitivity 0.60, F1 score 0.52, P_i_ 0.47). In this video, anxiety was confused with fear (33%), disgust (5%) or sadness (3%). The same video showing facial expressions was correctly recognized by 75% of participants, while anxiety was confused for fear (22%), disgust (3%) or sadness (1%). Blurred video was correctly recognized significantly less frequently than the one showing facial expressions (*p* = 0.03).

#### Disgust

Video *Disgust 1* blurred was correctly recognized by 38% of participants (specificity 0.98, sensitivity 0.38, F1 score 0.48, P_i_ 0.35). In this video, disgust was confused with fear (44%), anxiety (15%), neutral (3%) or surprise (1%). The same video showing facial expressions was correctly recognized by 91% of participants, while disgust was confused for fear (6%), neutral (2%) or anxiety (1%). Blurred video was correctly recognized significantly less frequently than the one showing facial expressions (*p* < 0.001).

Video *Disgust 2* blurred was correctly recognized by 45% of participants (specificity 0.98, sensitivity 0.45, F1 score 0.55, P_i_ 0.34). In this video, disgust was confused with anxiety (30%), fear (23%) or neutral (2%). The same video showing facial expressions was correctly recognized by 95% of participants, while disgust was confused for fear (4%) or anxiety (1%). Blurred video was correctly recognized significantly less frequently than the one showing facial expressions (*p* < 0.001).

#### Sadness

Video *Sadness 1* blurred was correctly recognized by 87% of participants (specificity 0.98, sensitivity 0.91, F1 score 0.84, P_i_ 0.77). In this video, sadness was confused with neutral (10%), disgust (2%) or fear (1%). The same video showing facial expressions was correctly recognized by 97% of participants, while 3% confused sadness for neutral. Blurred video was correctly recognized significantly less frequently than the one showing facial expressions (*p* = 0.01).

Video *Sadness 2* blurred was correctly recognized by 100% of participants (specificity 0.98, sensitivity 1, F1 score 0.89, P_i_ 1). The same video showing facial expressions was correctly recognized by 100% of participants.

#### Anger

Video *Anger 1* blurred was correctly recognized by 92% of participants (specificity 0.99, sensitivity 0.92, F1 score 0.94, P_i_ 0.84). In this video, anger was confused with neutral (4%), disgust (1%), anxiety (1%), happiness (1%) or sadness (1%). The same video showing facial expressions was correctly recognized by 99% of participants, while 1% confused anger with disgust. Blurred video was correctly recognized significantly less frequently than the one showing facial expressions (*p* = 0.02).

Video *Anger 2* blurred was correctly recognized by 98% of participants (specificity 0.99, sensitivity 0.98, F1 score 0.97, P_i_ 0.96). In this video, anger was confused with disgust (1%) or anxiety (1%). The same video showing facial expressions was correctly recognized by 98% of participants, while 2% confused anger with disgust.

### Emotion valence

**Fig. 3:**
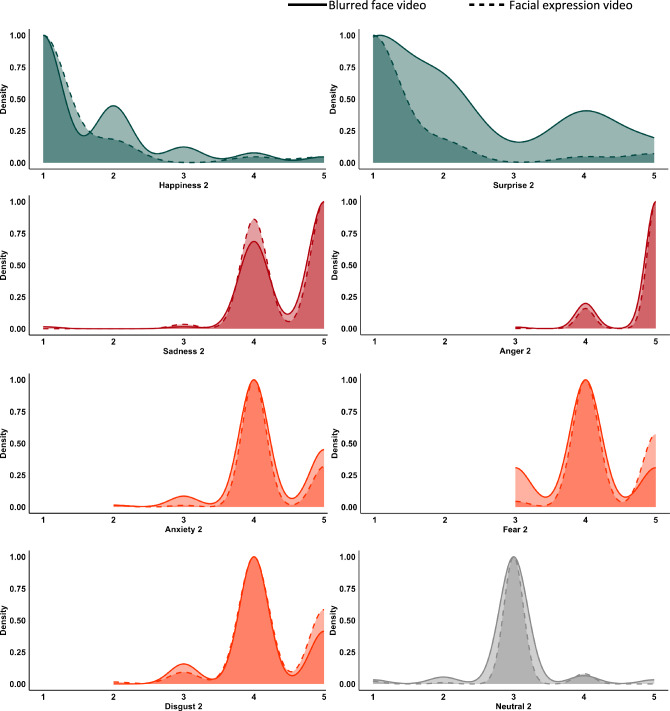
Kernel density estimates representing the distribution of valence scores associated with each video for both blurred-face conditions (bold line) and videos showing facial expressions (dashed line). Density represents the number of subjects answering a score, normalized in a range 0 to 1. Colors represent different valence scores: dark green = extremely positive (score 1), light green = positive (score 2), grey = neutral (score 3), orange = negative (score 4) and red = extremely negative (score 5)

Table 1 reports the detailed frequency of valence scores associated by the subjects to each video. Figure 3 and Supplementary Fig. S1 represent the distribution of valence scores associated with each video. For both blurred videos and videos showing facial expressions, neutral gait was generally associated with neutral valence while participants attributed negative valence to the emotions sadness, fear, anxiety, anger and disgust (only videos showing facial expressions). Happiness and surprise were evaluated as positive. The valence attributed to disgust in blurred videos cannot be considered valid considering the low rate of correct emotion recognition. Valence scores were significantly different between blurred videos and videos showing facial expressions for Happiness 1 and 2, Fear 1 and 2, Anger 1, Disgust 1 and Surprise 1 and 2 (Table 1).

### Emotion intensity

**Fig. 4:**
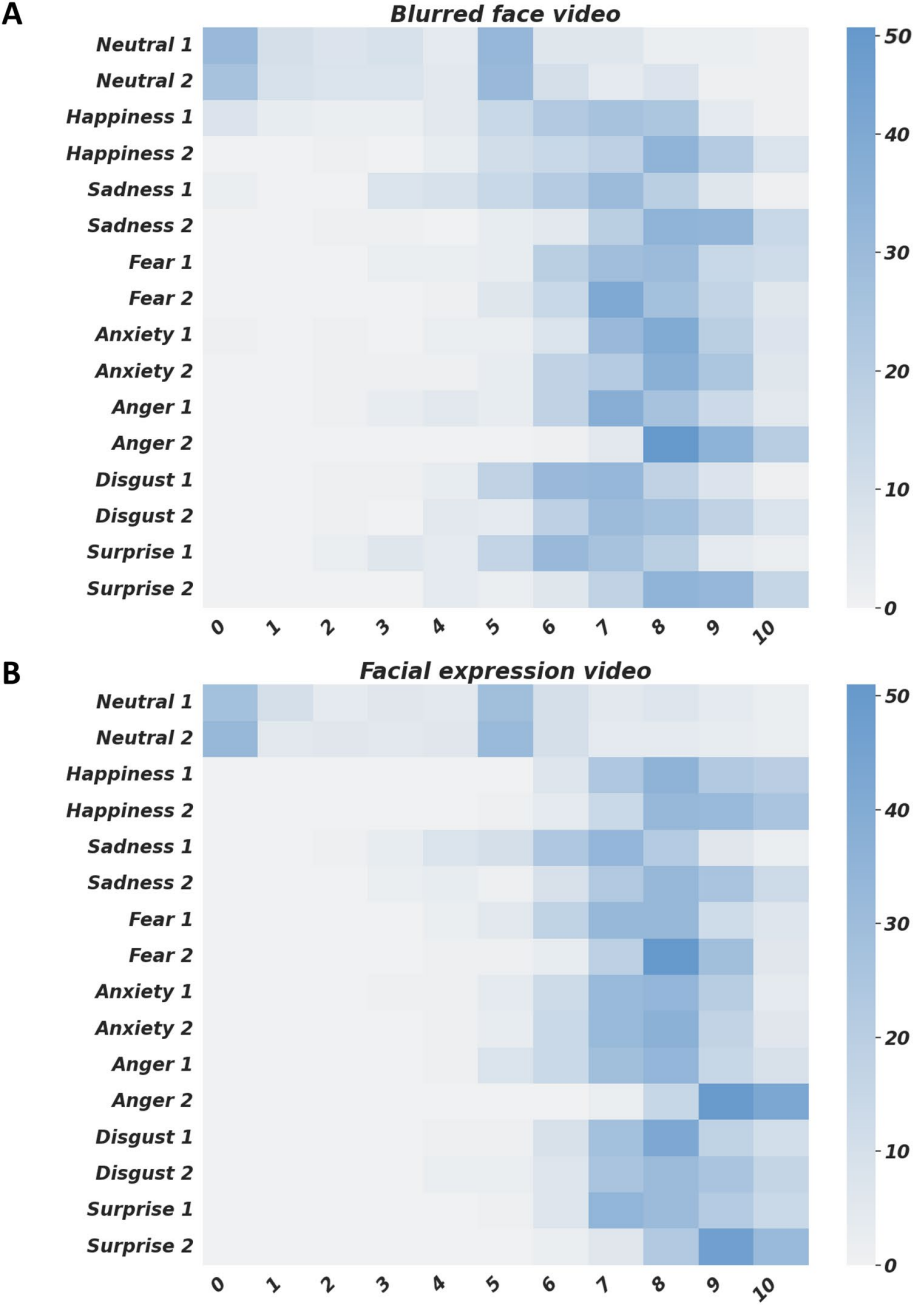
Distribution of intensity scores both in blurred-face videos (**A**) and in videos showing facial expressions (**B**). Intensity ranged from 0 to 10 (0 = no intensity, 10 = maximal intensity). Color bar represents the number of subjects attributing each specific intensity level

Table 1 reports mean and standard deviation values of the intensity associated to the emotions in each video. Figure 4 represents the distribution of intensity scores associated with each video. Generally, all the emotions more frequently correctly recognized, except for the neutral video, were rated around 7 or 8 on a scale ranging from 0 to 10, both in blurred videos and videos showing facial expressions. The neutral condition showed low perceived intensity (about 3 out of 10). Perceived intensity was significantly lower in blurred videos relative to videos showing facial expressions in Happiness 1 and 2, Fear 2, Anger 2, Disgust 1 and 2, Surprise 1 and 2 (Table 1).

### Selected videos

Based on our findings regarding emotion recognition, intensity, and valence, we selected one effective blurred video for each emotion (except for disgust and surprise) as follows: Happiness (video 2), Sadness (video 2), Anxiety (video 1, also suitable for evoking fear), Fear (video 2), and Anger (video 2). These stimuli may be valuable for future studies investigating emotion recognition and embodied simulation in patients with neurological conditions such as PD.

## Discussion

### Summary of main findings

The present study aimed to assess whether healthy adults can recognize emotional states expressed through gait in the absence of facial cues, and to identify a set of validated gait videos suitable for future applications in clinical populations, particularly patients with PD. Our findings support the hypothesis that emotions can be conveyed and recognized through body movement alone, although with varying degrees of accuracy, specificity and sensitivity depending on the specific emotion. This work represents an important preliminary step toward the development of ecologically valid, embodied emotional stimuli that can be used to probe affective processing and motor-affective integration in both health and disease conditions.

One of the most important results was that several emotions, namely neutral, happiness, sadness, fear/anxiety, and anger, were recognized with high accuracy, specificity and sensitivity from gait patterns even when facial information was removed. Moreover, we found a substantial Fleiss’ κ and high P_i_ values indicating that participants showed a high level of agreement in recognizing the emotions conveyed by gait. This suggests that emotional information was reliably expressed and perceived through movement patterns, supporting the validity of the stimuli used in the study. In some cases (e.g., Neutral 1), recognition rates were even higher in the blurred-face condition than when facial expressions were visible. This may indicate that facial features can sometimes interfere with, rather than aid, the decoding of body-language-based emotional cues. This observation aligns with the bottom-up embodiment theory, which suggests that emotional understanding arises from sensorimotor simulation of observed movements [29, 30], rather than solely from cognitive processing of facial or verbal cues [31].

### Emotion-specific challenges

However, not all emotions were equally well recognized. Disgust proved particularly challenging to identify based on gait alone, with recognition rates falling below 50% in certain videos. These findings are consistent with previous research indicating that some emotions, particularly disgust, rely more heavily on facial musculature (e.g., nose wrinkling, upper lip retraction), and less on whole-body expression [32]. Conversely, sadness and anger have been shown to involve consistent postural correlates (e.g., slumped shoulders, reduced arm swing, short strides and increased trunk rigidity), which may facilitate their detection even in the absence of facial information [6, 7, 33].

The frequent confusion between fear and anxiety is also noteworthy and has been previously reported in the affective neuroscience literature [34]. Both emotions share overlapping physiological substrates, such as hyperarousal, and similar motor features, including reduced stride length, increased body tension, and altered arm swing [15, 35, 36]. From a clinical perspective, the overlap between fear and anxiety highlights that their distinction can only be made when considering the context in which these emotions are experienced. For example, in the case of fear, it is crucial that the eliciting stimulus is clearly present and identifiable (e.g., encountering a growling wolf). In contrast, anxiety typically arises from anticipatory cognitive processes, often involving more or less catastrophic thoughts about the future. In the proposed video stimuli, these contextual elements are absent; therefore, in the absence of such information, we suggest treating fear and anxiety as a composite category when analyzing embodied emotion recognition.

### Valence and intensity perception

The valence and intensity ratings provided additional insights into the way emotions are perceived through gait. As expected, neutral gait was consistently rated as emotionally neutral and of low intensity, while happiness and surprise were perceived as positive and moderately intense, and emotions like sadness, fear, anger, and anxiety were rated as negative with moderate to high intensity. Importantly, the valence and intensity of blurred-face videos often differed significantly from those including facial expressions, highlighting the influence of facial information to amplify emotional perception [31]. Nevertheless, several blurred-face videos still elicited coherent emotional ratings, suggesting that gait alone can carry sufficient information for the perception of emotional intensity and valence in many cases [3–5, 8].

### Clinical implications

The clinical implications of these findings are significant. In recent years, growing attention has been given to the bidirectional relationship between emotion and movement [13]. In PD, emotional state has been shown to influence gait parameters: for instance, negative emotions correlate with slower walking speed, reduced stride length, and forward trunk flexion, while positive emotions are linked to increased walking speed and more upright posture [13, 37, 38]. Emotional states can influence motor parameters, and conversely, motor patterns may reinforce or modulate affective experience through embodied feedback mechanisms. The reciprocal relationship between motor behavior and emotional state suggests that emotions could be exploited therapeutically to enhance gait function, particularly in patients with motor and affective impairments. This idea aligns with embodied cognition theories, which posit that movement and emotion are interdependent processes [31]. For example, adopting a joyful gait posture, characterized by upright trunk, rhythmic arm swing, and increased stride length, could potentially trigger positive affect via feedback loops between somatic expression and emotional regulation [15, 39, 40]. This suggests that emotionally-informed gait training, where patients are encouraged to embody positive emotional states through movement (e.g., adopting an upright posture, rhythmic arm swing), could be a novel approach to motor rehabilitation promoting both motor and affective benefits. The validation of emotionally expressive gait stimuli in healthy adults is a necessary first step toward implementing such interventions.

### Future directions

One potential avenue is the integration of these validated stimuli into AOT protocols. AOT, which involves observing goal-directed actions to facilitate motor learning via MNS activation, has shown efficacy in improving gait in PD and other motor disorders [21–27]. To date, however, AOT paradigms have generally relied on neutral motor actions. The addition of emotional content to these actions, tested in healthy populations for recognition accuracy, specificity, sensitivity, valence, and intensity, could enhance engagement and learning by stimulating emotional-motor circuits resonance and maximize the effectiveness of AOT paradigms [41]. Moreover, the fact that certain emotions such as happiness and sadness were reliably recognized even in blurred-face videos suggests that bottom-up processes of embodiment are sufficient to trigger affective understanding. This is particularly relevant for individuals with deficits in cognitive-emotional processing (as in some forms of frontotemporal dementia or in advanced PD), where access to abstract or verbal emotional cues may be impaired. Presenting emotional content through movement could offer an alternative channel for communication and therapeutic engagement, allowing emotionally meaningful stimuli to bypass higher-order deficits and reach affective-motor systems through a more embodied route [29–31]. Future research could investigate whether AOT protocols that incorporate gait videos with distinct emotional content, rather than neutral movements, produce greater improvements in spatio-temporal gait parameters in individuals with neurological disorders. By engaging both motor and affective systems, emotionally enriched gait observation might not only enhance motor performance but also support emotional-motor coupling, offering a novel approach to rehabilitation.

### Limitations

Several limitations of this study warrant consideration. First, we used a single actress for all gait recordings. While this allowed for control over stylistic variation, it may limit generalizability, as emotional expression is highly individual. For instance, previous evidence suggested that motor resonance, the process that supports emotion understanding through action observation, can be affected by gender similarity between the observer and the actor. Thus, future studies should include multiple actors of different genders, ages, and body types to enhance the robustness and external validity of the results. Second, we did not collect data on the participants’ own emotional states at the time of testing, which could influence perception. Given that emotional perception is modulated by mood congruency future versions should incorporate mood assessments [42]. Third, the use of a treadmill for gait recordings, while ensuring standardized video production, may limit natural movement and emotional expressivity compared to overground walking. Treadmill walking can modify gait parameters such as cadence and stride length, reducing the spontaneity typical of real-world gait. Future studies should include overground or more ecological conditions to validate and extend the current findings. Fourth, our results provide a basis for stratified analysis in future studies. While our current sample included healthy adults in their emotional and motor maturity (ages 25–40), the emotional salience of these gait patterns should be tested in older adults and in neurological populations such as PD. This would determine whether the selected videos are robust to age-related perceptual or motor changes, and whether emotion recognition accuracy correlates with motor severity or mood disturbances in PD.

## Conclusions

In conclusion, this study provides preliminary validation of a set of emotional gait videos as reliable stimuli for emotion recognition in healthy adults, confirming that gait alone can convey discrete emotional states. The validated stimuli, especially those exceeding 90% recognition accuracy and achieving high sensitivity and specificity scores, can be utilized in future research about emotional embodiment in neurological diseases such as PD. By first validating emotional gait stimuli in a healthy population, we established a robust methodological foundation for investigating how individuals with neurological conditions perceive and embody these emotional signals. This two-step approach ensures that future studies and interventions are grounded in ecologically valid and perceptually reliable materials. The integration of emotional cues into gait observation and training represents a promising and innovative direction for both research and rehabilitation, offering both theoretical advancements in emotion–motor coupling and practical applications exploiting mirror neuron and limbic system mechanisms. Ultimately, by combining insights from embodied cognition, affective neuroscience, and motor rehabilitation, this line of research holds the potential to inform emotionally enriched therapeutic strategies that might enhance not only motor function but also emotional engagement and overall quality of life.

## Supplementary Information

Below is the link to the electronic supplementary material.

Supplementary file 1 (DOCX 19 KB)

Supplementary file 2 (PPTX 852 KB)

## Data Availability

The dataset used and analyzed during the current study is available from the corresponding author upon request to qualified researchers (i.e., affiliated to a university or research institution/hospital).

## References

[1] Barliya A, Omlor L, Giese MA, Berthoz A, Flash T (2013) Expression of emotion in the kinematics of locomotion. Exp Brain Res 225:159–176 23250443 10.1007/s00221-012-3357-4

[2] Noroozi F, Corneanu C, D K, T S, S E, S A (2021) Survey on emotional body gesture recognition. IEEE Trans Affect Comput 12:505–523

[3] Riemer H, Joseph JV, Lee AY, Riemer R (2023) Emotion and motion: toward emotion recognition based on standing and walking. PLoS ONE 18:e0290564 10.1371/journal.pone.0290564 PMC10499259 37703239

[4] Atkinson AP, Dittrich WH, Gemmell AJ, Young AW (2004) Emotion perception from dynamic and static body expressions in point-light and full-light displays. Perception 33:717–746 15330366 10.1068/p5096

[5] Gross MM, Crane EA, Fredrickson BL (2012) Effort-shape and kinematic assessment of bodily expression of emotion during gait. Hum Mov Sci 31:202–221 21835480 10.1016/j.humov.2011.05.001

[6] Halovic S, Kroos C (2018) Not all is noticed: kinematic cues of emotion-specific gait. Hum Mov Sci 57:478–488 29174557 10.1016/j.humov.2017.11.008

[7] Melzer A, Shafir T, Tsachor RP (2019) How do we recognize emotion from movement? Specific motor components contribute to the recognition of each emotion. Front Psychol 10:1389 31333524 10.3389/fpsyg.2019.01389 PMC6617736

[8] Roether CL, Omlor L, Christensen A, Giese MA (2009) Critical features for the perception of emotion from gait. J Vis 9(15):11–32 19761306 10.1167/9.6.15

[9] Reed CL, Moody EJ, Mgrublian K, Assaad S, Schey A, McIntosh DN (2020) Body matters in emotion: restricted body movement and posture affect expression and recognition of status-related emotions. Front Psychol 11:1961 32849150 10.3389/fpsyg.2020.01961 PMC7432155

[10] Rizzolatti G, Sinigaglia C (2010) The functional role of the parieto-frontal mirror circuit: interpretations and misinterpretations. Nat Rev Neurosci 11:264–274 20216547 10.1038/nrn2805

[11] Rizzolatti G, Sinigaglia C (2016) The mirror mechanism: a basic principle of brain function. Nat Rev Neurosci 17:757–765 27761004 10.1038/nrn.2016.135

[12] Schneider S, Christensen A, Haussinger FB, Fallgatter AJ, Giese MA, Ehlis AC (2014) Show me how you walk and I tell you how you feel—a functional near-infrared spectroscopy study on emotion perception based on human gait. Neuroimage 85:380–390 23921096 10.1016/j.neuroimage.2013.07.078

[13] Avanzino L, Lagravinese G, Abbruzzese G, Pelosin E (2018) Relationships between gait and emotion in Parkinson’s disease: a narrative review. Gait Posture 65:57–64 30558947 10.1016/j.gaitpost.2018.06.171

[14] Lagravinese G, Pelosin E, Bonassi G, Carbone F, Abbruzzese G, Avanzino L (2018) Gait initiation is influenced by emotion processing in Parkinson’s disease patients with freezing. Mov Disord 33:609–617 29392774 10.1002/mds.27312

[15] Michalak J, Troje NF, Fischer J, Vollmar P, Heidenreich T, Schulte D (2009) Embodiment of sadness and depression–gait patterns associated with dysphoric mood. Psychosom Med 71:580–587 19414617 10.1097/PSY.0b013e3181a2515c

[16] Mirelman A, Bonato P, Camicioli R, Ellis TD, Giladi N, Hamilton JL, Hass CJ, Hausdorff JM, Pelosin E, Almeida QJ (2019) Gait impairments in Parkinson’s disease. Lancet Neurol 18(7):697–708. 10.1016/S1474-4422(19)30044-4 30975519 10.1016/S1474-4422(19)30044-4

[17] Sarasso E, Filippi M, Agosta F (2023) Clinical and MRI features of gait and balance disorders in neurodegenerative diseases. J Neurol 270:1798–1807 36577818 10.1007/s00415-022-11544-7

[18] Homagain A, Ehgoetz Martens KA (2023) Emotional states affect steady state walking performance. PLoS ONE 18:e0284308 37708145 10.1371/journal.pone.0284308 PMC10501668

[19] Kang GE, Gross MM (2015) Emotional influences on sit-to-walk in healthy young adults. Hum Mov Sci 40:341–351 25681657 10.1016/j.humov.2015.01.009

[20] Ernst M, Folkerts AK, Gollan R et al (2023) Physical exercise for people with Parkinson’s disease: a systematic review and network meta-analysis. Cochrane Database Syst Rev 1:CD013856 36602886 10.1002/14651858.CD013856.pub2 PMC9815433

[21] Abbruzzese G, Avanzino L, Marchese R, Pelosin E (2015) Action observation and motor imagery: innovative cognitive tools in the rehabilitation of Parkinson’s disease. Parkinsons Dis 2015:124214 26495150 10.1155/2015/124214 PMC4606219

[22] Agosta F, Gatti R, Sarasso E et al (2017) Brain plasticity in Parkinson’s disease with freezing of gait induced by action observation training. J Neurol 264:88–101 27778161 10.1007/s00415-016-8309-7

[23] Bommarito G, Putzolu M, Avanzino L, Cosentino C, Botta A, Marchese R, Inglese M, Pelosin E (2020) Functional correlates of action observation of gait in patients with Parkinson’s disease. Neural Plast 2020:8869201. 10.1155/2020/8869201 33456457 10.1155/2020/8869201 PMC7787806

[24] Pelosin E, Avanzino L, Bove M, Stramesi P, Nieuwboer A, Abbruzzese G (2010) Action observation improves freezing of gait in patients with Parkinson’s disease. Neurorehabil Neural Repair 24:746–752 20453155 10.1177/1545968310368685

[25] Sarasso E, Agosta F, Piramide N, Canu E, Volonte MA, Filippi M (2021) Brain activity of the emotional circuit in Parkinson’s disease patients with freezing of gait. Neuroimage Clin 30:102649 33838547 10.1016/j.nicl.2021.102649 PMC8045031

[26] Sarasso E, Agosta F, Piramide N, Gardoni A, Canu E, Leocadi M, Castelnovo V, Basaia S, Tettamanti A, Volontè MA, Filippi M (2021) Action observation and motor imagery improve dual task in Parkinson’s disease: a clinical/fMRI study. Mov Disord 36(11):2569–2582. 10.1002/mds.28717 34286884 10.1002/mds.28717

[27] Sarasso E, Gardoni A, Zenere L, Canu E, Basaia S, Pelosin E, Volontè MA, Filippi M, Agosta F (2023) Action observation and motor imagery improve motor imagery abilities in patients with Parkinson’s disease—a functional MRI study. Parkinsonism Relat Disord 116:105858. 10.1016/j.parkreldis.2023.105858 37774517 10.1016/j.parkreldis.2023.105858

[28] Stephanou K, Davey CG, Kerestes R et al (2016) Brain functional correlates of emotion regulation across adolescence and young adulthood. Hum Brain Mapp 37:7–19 26596970 10.1002/hbm.22905 PMC6867496

[29] Gallese V, Sinigaglia C (2011) What is so special about embodied simulation? Trends Cogn Sci 15:512–519 21983148 10.1016/j.tics.2011.09.003

[30] Gallese Vittorio SC (2018) Embodied resonance. The Oxford handbook of 4E cognition. Oxford University Press, Oxford, pp 417–432

[31] Niedenthal PM (2007) Embodying emotion. Science 316:1002–1005 17510358 10.1126/science.1136930

[32] Rozin P, Haidt, J., & McCauley, C. R. Disgust. Handbook of emotions 2008;The Guilford Press:757–776.

[33] Li S, Cui L, Zhu C, Li B, Zhao N, Zhu T (2016) Emotion recognition using Kinect motion capture data of human gaits. PeerJ 4:e2364 27672492 10.7717/peerj.2364 PMC5028730

[34] Sylvers P, Lilienfeld SO, LaPrairie JL (2011) Differences between trait fear and trait anxiety: implications for psychopathology. Clin Psychol Rev 31:122–137 20817337 10.1016/j.cpr.2010.08.004

[35] Dunsmoor JE, Paz R (2015) Fear generalization and anxiety: behavioral and neural mechanisms. Biol Psychiatry 78:336–343 25981173 10.1016/j.biopsych.2015.04.010

[36] Tovote P, Fadok JP, Luthi A (2015) Neuronal circuits for fear and anxiety. Nat Rev Neurosci 16:317–331 25991441 10.1038/nrn3945

[37] Naugle KM, Hass CJ, Bowers D, Janelle CM (2012) Emotional state affects gait initiation in individuals with Parkinson’s disease. Cogn Affect Behav Neurosci 12:207–219 22194236 10.3758/s13415-011-0071-9 PMC3618281

[38] Naugle KM, Hass CJ, Joyner J, Coombes SA, Janelle CM (2011) Emotional state affects the initiation of forward gait. Emotion 11:267–277 21500896 10.1037/a0022577

[39] Goldberg H, Christensen A, Flash T, Giese MA, Malach R (2015) Brain activity correlates with emotional perception induced by dynamic avatars. Neuroimage 122:306–317 26220746 10.1016/j.neuroimage.2015.07.056

[40] Wu YT, Baillet S, Lamontagne A (2024) Brain mechanisms involved in the perception of emotional gait: a combined magnetoencephalography and virtual reality study. PLoS ONE 19:e0299103 38551903 10.1371/journal.pone.0299103 PMC10980214

[41] Calvo-Merino B, Glaser DE, Grezes J, Passingham RE, Haggard P (2005) Action observation and acquired motor skills: an FMRI study with expert dancers. Cereb Cortex 15:1243–1249 15616133 10.1093/cercor/bhi007

[42] Schmid PC, Schmid Mast M (2010) Mood effects on emotion recognition. Motiv Emot 34(3):288–292. 10.1007/s11031-010-9170-0

