# Supplementary material for: The language of gait: interpreting emotional states through gait videos
Source: J Neurol. 2025 Nov 18;272(12):770. doi: 10.1007/s00415-025-13519-w (PMC12627203; doi:10.1007/s00415-025-13519-w)
Supplement: Supplementary file 1 — (DOCX 19 KB) [file 415_2025_13519_MOESM1_ESM.docx]

**Supplementary table 1.** Specificity, sensibility, F1 and Pᵢ scores for each blurred face video.

| **Blurred face video** | **Specificity** | **Sensitivity** | **F1 score** | **Pᵢ** |
| --- | --- | --- | --- | --- |
| ***Neutral 1*** | **0.96** | **0.98** | **0.75** | **0.96** |
| *Neutral 2* | 0.96 | 0.88 | 0.70 | 0.79 |
| *Happiness 1* | 0.99 | 0.66 | 0.75 | 0.55 |
| ***Happiness 2*** | **0.99** | **0.91** | **0.90** | **0.83** |
| ***Surprise 1*** | **0.99** | **0.85** | **0.87** | **0.73** |
| *Surprise 2* | 0.99 | 0.85 | 0.87 | 0.72 |
| *Fear 1* | 0.93 | 0.84 | 0.57 | 0.71 |
| ***Fear 2*** | **0.93** | **0.91** | **0.60** | **0.83** |
| ***Anxiety 1*** | **0.95** | **0.85** | **0.67** | **0.74** |
| *Anxiety 2* | 0.95 | 0.60 | 0.52 | 0.47 |
| *Disgust 1* | 0.98 | 0.38 | 0.48 | 0.35 |
| *Disgust 2* | 0.98 | 0.45 | 0.55 | 0.34 |
| *Sadness 1* | 0.98 | 0.91 | 0.84 | 0.77 |
| ***Sadness 2*** | **0.98** | **1.00** | **0.89** | **1.00** |
| *Anger 1* | 0.99 | 0.92 | 0.94 | 0.84 |
| ***Anger 2*** | **0.99** | **0.98** | **0.97** | **0.96** |

*Specificity* indicates the proportion of videos that did not express a given emotion and were correctly identified as not belonging to that emotional category; *sensitivity* is the proportion of videos that actually express a given emotion and were correctly recognized by participants; *F1 score* is a combined metric that captures a balance between precision and sensitivity, evaluating how well participants both detect the correct emotion and avoid false positives; *Pᵢ* represents the degree of agreement among raters for a single video.
