# Supplementary material for: The language of gait: interpreting emotional states through gait videos
Source: J Neurol. 2025 Nov 18;272(12):770. doi: 10.1007/s00415-025-13519-w (PMC12627203; doi:10.1007/s00415-025-13519-w)
Supplement: Supplementary file 2 — (PPTX 852 KB) [file 415_2025_13519_MOESM2_ESM.pptx]

## Slide 1
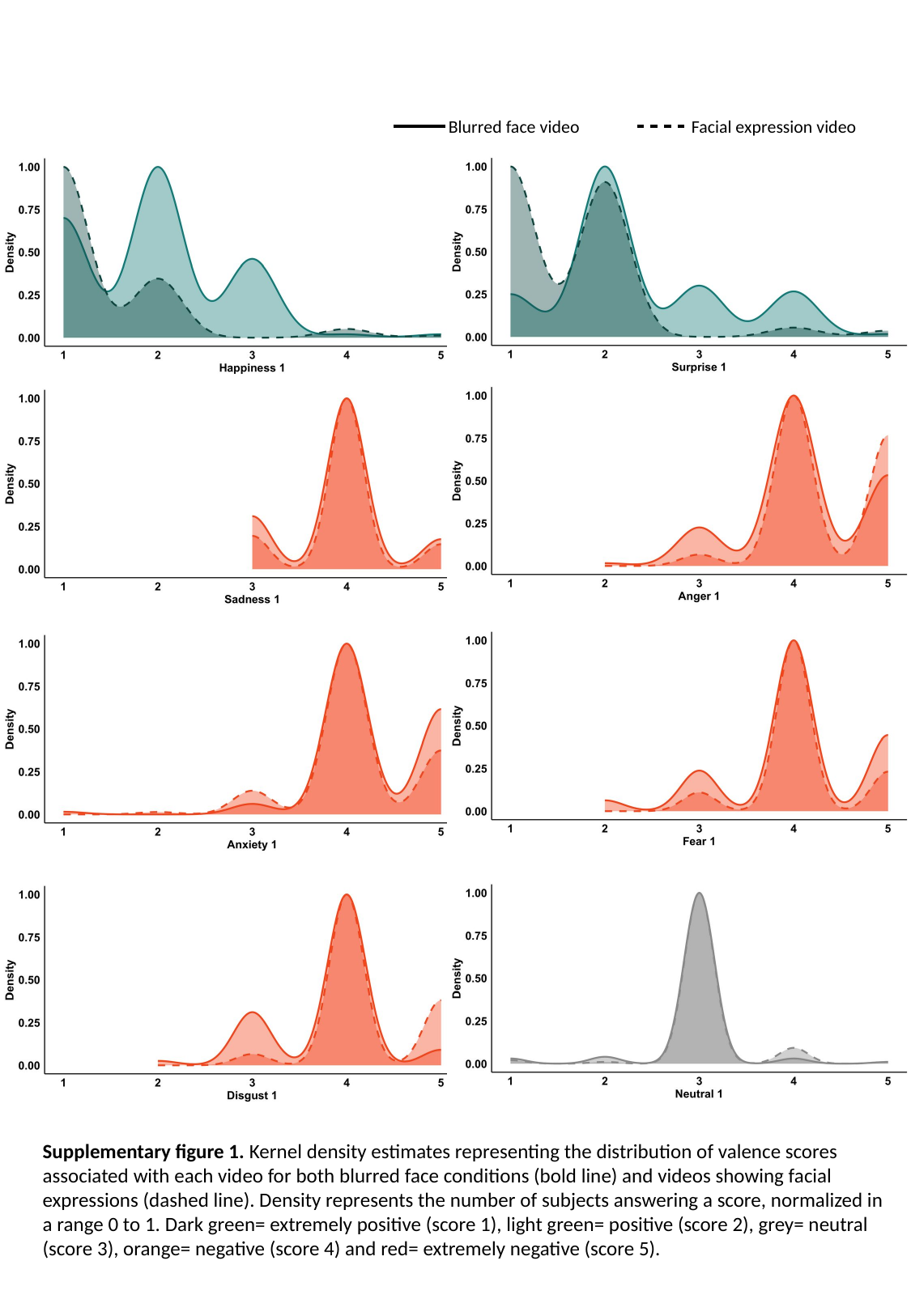

Blurred face video
Facial expression video
Supplementary figure 1. Kernel density estimates representing the distribution of valence scores associated with each video for both blurred face conditions (bold line) and videos showing facial expressions (dashed line). Density represents the number of subjects answering a score, normalized in a range 0 to 1. Dark green= extremely positive (score 1), light green= positive (score 2), grey= neutral (score 3), orange= negative (score 4) and red= extremely negative (score 5).
